# Disease quiescence in endophthalmitis patients treated with anti-VEGF injections for retinal pathologies

**DOI:** 10.1186/s12886-024-03336-6

**Published:** 2024-02-16

**Authors:** Brice Nguedia Vofo, Majd Saada, Antonio Rivera, Sigalit Cohen, Tareq Jaouni, Samer Khateb

**Affiliations:** Department of Ophthalmology, Hadassah Medical Center, Faculty of Medicine, The Hebrew University of Jerusalem, 91120 Jerusalem, POB 12000, Israel

**Keywords:** Endophthalmitis, Intravitreal injections, Age-related macular degeneration, Myopic choroidal neovascularization, Retinal vein occlusion, Anti-vascular endothelial growth factor

## Abstract

**Background:**

The most feared complication of intravitreal injections is the development of endophthalmitis, which could lead to irreversible visual loss. The aim of this study was to characterize the clinical profiles, causative pathogens, and clinical outcome of patients post-endophthalmitis.

**Methods:**

Retrospective, single center case series study. Clinical records, causative pathogens and management of all cases of endophthalmitis post intravitreal anti-vascular endothelial growth factor (VEGF) injections recorded between January 1st, 2006 and May 30th, 2022; were retrieved. The visual and anatomic changes prior to the episode of endophthalmitis and up to 2 years post-treatment were compared.

**Results:**

Eleven post-injection endophthalmitis eyes of 10 patients (*n* = 3 females; 30%) were recruited at mean age of 64.5 ± 20.4 years. The median last recorded BCVA, up to 3 months prior to the episode of endophthalmitis was 60 (Interquartile range (IQR) 55–75) ETDRS letters. Then, it dropped to 30 (IQR 0-57.5), 35 (IQR 0-52.5) and 35 (IQR 0-57.5) ETDRS letters at presentation, 6- and 12-months follow-up; respectively (*p* = 0.027, *p* = 0.017 and *p* = 0.012). However, at 24 months, the median BCVA returned to similar baseline values prior to the episode of endophthalmitis; BCVA 50 (IQR 0–60) ETDRS letters, *p* = 0.062. Interestingly, two eyes with neovascular age-related macular degeneration (NVAMD), 1 with myopic choroidal neovascularization (CNV) and 1 with retinal vein occlusion (RVO), experienced disease quiescence and did not require additional anti-VEGF injections up to 2 years of follow-up.

**Conclusion:**

This study demonstrates long-term recovery of vision loss due to endophthalmitis post anti-VEGF injections, regained up to 2 years later. It also indicates that disease quiescence post endophthalmitis may not only occur in eyes treated for NVAMD, but also with myopic CNV and RVO.

## Introduction

Over the last two decades, there has been an exponential increase in the use of intravitreal injections of anti-vascular endothelial growth factors (VEGF) compounds in the management of a variety of retinal pathologies [1]. The most feared complication of this procedure is the development of endophthalmitis, which could lead to irreversible visual loss [2, 3]. A meta-analysis reported an incidence of about one endophthalmitis case for every 1779 injections of anti-VEGF (0.056%).^4^ The most common pathogens identified in culture are staphylococcus and streptococcus species [4]. However, several previous reports described quiescence of disease activity in neovascular age related macular degeneration (NVAND) patients, post-endophthalmitis, [5–12] and one case with myopic choroidal neovascularization (CNV) [11]. The exact reason for this quiescence is unknown. Current hypothesis include a possible decrease in the proangiogenic cytokines due to endophthalmitis, which could induce a state of vascular remodeling that contributes to the stabilization of existing CNV [13]. Another plausible explanation has been the decrease oxygen demand and angiogenic drive, following the extensive tissue damage due to severe inflammation, [5] or rather the disrupted blood-ocular barrier leading to long term changes in intraocular cytokine profiles that may modulate CNV activity [6]. 

Understanding the relationship between anti-VEGF treatment and endophthalmitis can help clinicians make informed decisions about the management of patients with retinal pathologies, and potentially reduce the risk of severe visual loss associated with these treatments. Its currently known that the use of pre-injection povidone-iodine reduces the occurrence of post-injection endophthalmitis, [14] however, no benefit has been seen, in the use of pre-injection or post-injection topical antibiotics [15]. 

This disease quiescence after endophthalmitis has also been observed amongst a several cases post endophthalmitis in our clinic. The objective of this study is therefore to characterize the clinical profiles, causative pathogens, and clinical outcome of our patients post endophthalmitis.

## Methods

### Study design

A retrospective study of all cases with endophthalmitis registered between January 1^st,^ 2006 and May 30th, 2022 at the Hadassah Medical Center, Jerusalem, Israel. Eyes that were treated with intravitreal injections of anti-VEGF compound for NVAMD, diabetic macula edema (DME) and retinal vein occlusion (RVO) for which an episode of endophthalmitis was diagnosed and they stayed in follow-up for at least 1 year were included. If available, data for up to 2 years was collected to depict treatment continuation post endophthalmitis. Patients with endophthalmitis due to trauma, or patients treated elsewhere, were excluded.

### Data collection

Demographics and clinical data were collected retrospectively from the patients’ electronic medical records. Best-corrected visual acuity (BCVA) was routinely assessed using the Early Treatment for Diabetic Retinopathy Study (ETDRS) chart. The number, and frequency of intravitreal injections administered were recorded. The OCT data, up to 3 months before and after the episode of endophthalmitis, were collected using the Spectralis HRA-OCT (Heidelberg Engineering, Heidelberg, Germany). The central subfield thickness (CST, defined as the average thickness in the central 1-mm diameter) and maximum central subfield thickness (MCST, defined as the thickest section within the 9 ETDRS subfields) were both collected. Clinical notes on the type of intervention and the organisms identified on culture samples were also noted.

### Anti-VEGF treatment protocols

The patients are treated using the treat and extend protocol. In this protocol, patients are treated with 3 monthly injections as a loading dose. Thereafter, disease activity, defined by the presence of IRF, SRF, and/or a new subretinal macular hemorrhage, is used to guide the treatment interval to the next injection. In cases of disease quiescence or stability, extensions were gradually made; by no more than 2 weeks at a time. However, in cases of disease activity, treatment intervals could be shortened by more than 2 weeks at a time.

### Outcome measures

Outcomes included change in BCVA, CST, MCST post-endophthalmitis compared to values prior to endophthalmitis. The treatment intervals before and after the episodes of endophthalmitis, organisms identified on culture samples, and the rate of endophthalmitis amongst patients receiving anti-VEGF injections at the Hadassah medical center.

### Statistics

Statistical analyses were performed using SPSS, version 25.0 (IBM Corp., Armonk, NY). After testing for normality, groups were compared using a paired Student’s t-test or Wilcoxon signed-rank test, where appropriate. The prevalence of categorical parameters was compared using Fisher’s exact test. Differences were considered significant at *p* < 0.05. Except where indicated otherwise, summary data are presented as n (%) or mean ± SD.

## Results

### Patient characteristics

Eighteen patients (19 eyes) were suspected of having endophthalmitis. In 6 cases, the endophthalmitis occurred post-injection in another healthcare facility, and in 2 cases the final diagnosis was an inflammatory reaction and not endophthalmitis. In total, there were 11 post injections endophthalmitis eyes in 10 patients confirmed within the health facility amongst which 30% were female (*n* = 3) and the mean patient age at the time of the incident was 64.7 ± 21.1 years. Table 1 depicts more details on patient characteristics. In all cases, the antibiotic injected were Vancomycin 1 mg/0.1 ml and Ceftazidime 2.25 mg/0.1 ml. For cases 2 and 3, intravitreal dexamethasone 4 mg/0.1 was injected in addition to antibiotics during vitrectomy. Case 10 received 2 additional injections of vancomycin post-vitrectomy. In all cases complete vitrectomy was done as per the department’s protocol in cases of endophthalmitis. No tamponading agents (silicone oil or gas) were used in any case.

**Table 1:** Patient characteristics

No.	Gender	Background systemic condition	Laterality	Diagnosis	Anti-VEGF compound	BCVA at presentation	No. of Years on treatment before	Management	Vitreous sample culture growth
1	M		RE	Myopic CNV	Bevacizumab	HM	3	tap and inject + vitrectomy	Staphylococcus Epidermitis
2	M		RE	NVAMD	Aflibercept	0.08	4	tap and inject + vitrectomy	Staphylococcus Epidermitis
3	M	HTN, CHD	RE	RVO	Bevacizumab	HM	9	tap and inject + vitrectomy	Staphylococcus Epidermitis
4	F	HTN, CHD	LE	NVAMD	Ranibizumab	0.25	2	tap and inject	negative
5	M		RE	NVAMD	Bevacizumab	HM	5	tap and inject	negative
6	M		RE	NVAMD	Bevacizumab	HM	1	tap and inject + vitrectomy	negative
7	F	DMII	RE	DME	Aflibercept	0.5	4	tap and inject + vitrectomy	negative
8	M		LE	NVAMD	Ranibizumab	0.22	1	tap and inject + vitrectomy	Staphylococcus Epidermidis
9	F	DMII	LE	NVAMD	Ranibizumab	0.25	3	tap and inject	Staphylococcus Epidermidis
10	M	DMII, HTN	RE	DME	Aflibercept	0.3	1	tap and inject + vitrectomy	Staphylococcus Epidermidis
			LE	DME	Bevacizumab	CF	2	tap and inject + vitrectomy	Staphylococcus Epidermidis

Anti-VEGF, anti-vascular endothelial risk factor; BCVA, best-corrected visual acuity; M, male; F, female; RE, right eye; LE, left eye; HNT, hypertension; CHD, coronary heart disease; DMII, diabetes mellitus type 2; NVAMD, neovascular age related macular degeneration; RVO, retinal vein occlusion; DME, diabetic macular edema; CNV choroidal neovascularization; hand-motion, HM; counting fingers, CF

### Prevalence of endophthalmitis

Between January 1st, 2006, and May 30th, 2022, 130,195 intravitreal injections were administered. Eleven eyes of 10 patients developed endophthalmitis, resulting to a prevalence of 0.0085% (1 case of endophthalmitis per 11,836 intravitreal injections) of endophthalmitis per eye amongst patients receiving intravitreal injections for retinal pathologies.

### Visual acuity outcome

**Fig. 1:**
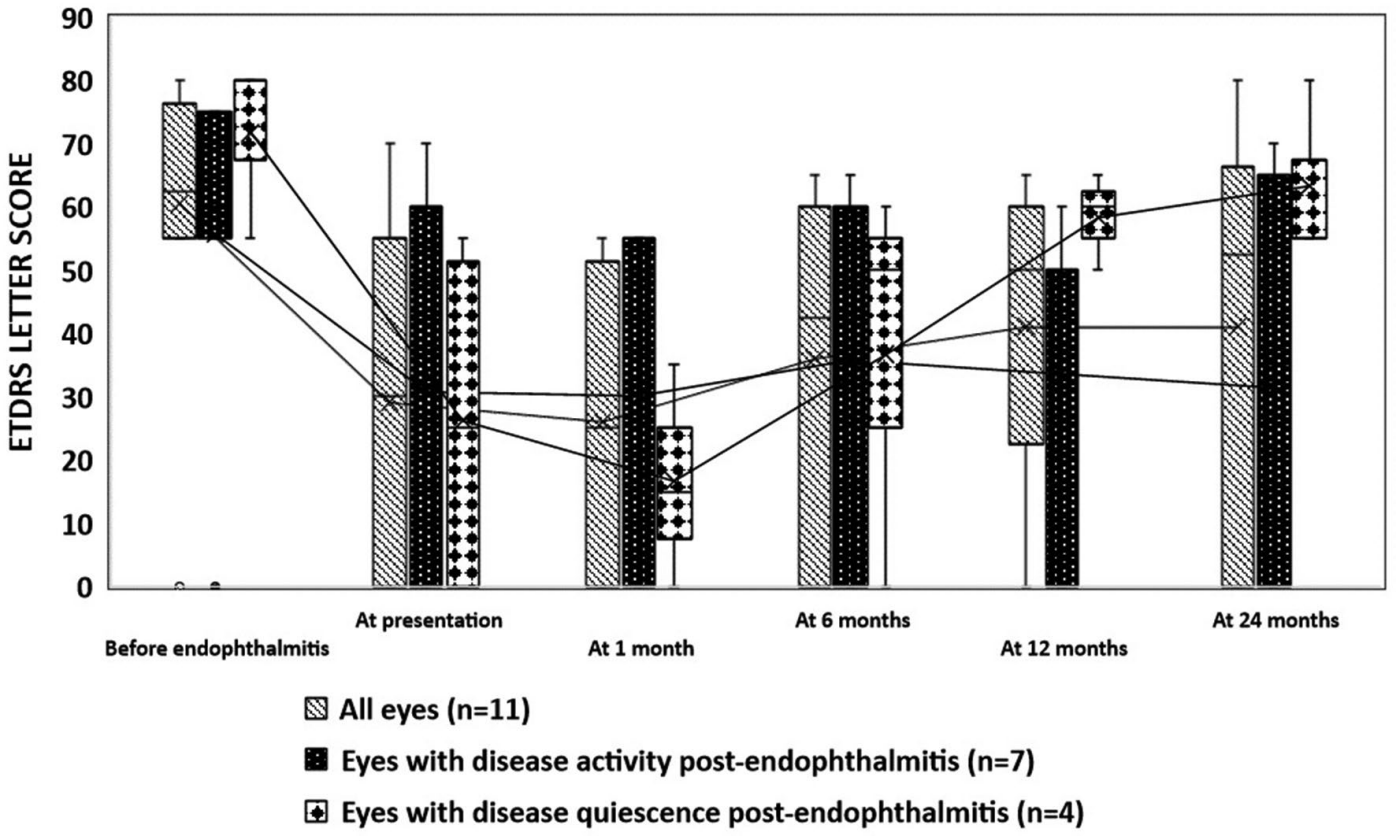
Box plot of BCVA ETDRS letter score before and after the episode of endophthalmitis in all eyes, eyes with disease activity and eyes with disease quiescence. * *p* < 0.05, compared to BCVA prior to endophthalmitis

The median last recorded BCVA, up to 3 months prior to the episode of endophthalmitis was 60 (Interquartile range (IQR) 55–75) ETDRS letters (mean LogMAR BCVA 0.45 ± 0.65). Then, it dropped to 30 (IQR 0-57.5) ETDRS letters (mean LogMAR BCVA 1.4 ± 0.85), 35 (IQR 0-52.5) ETDRS letters (mean LogMAR BCVA 1.2 ± 0.) and 35 (IQR 0-57.5) ETDRS letters (mean LogMAR BCVA 1.1 ± 0.9), at presentation, 6 months and 12 months follow-up; *p* = 0.027, *p* = 0.017 and *p* = 0.012, respectively. However, at 24 months follow-up, mean BCVA returned to baseline values prior to the episode of endophthalmitis; BCVA 50 (IQR 0–60) ETDRS letters (mean LogMAR BCVA 0.98 ± 0.8), *p* = 0.062. Figure 1 shows the boxplot of BCVA ETDRS letter score before and after the episode of endophthalmitis in all eyes, both with disease activity and with disease quiescence.

### Anatomical outcome

**Fig. 2:**
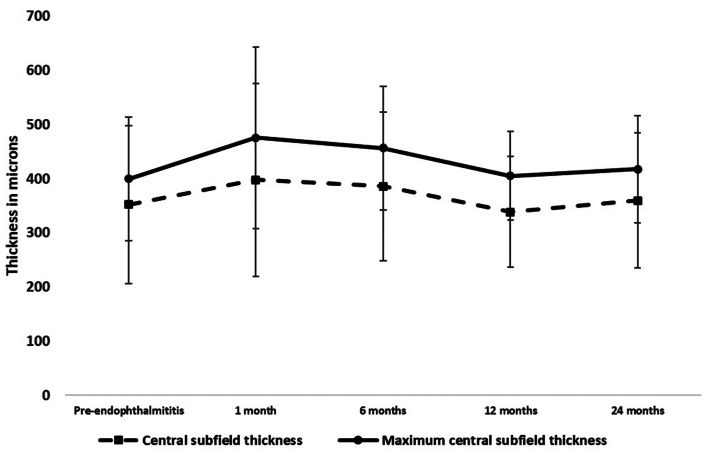
Central subfield thickness and maximum central subfield thickness change over time. Error bars represent standard deviation

The mean last CST and MCST prior to the episode of endophthalmitis was 352.1 ± 145.5 microns and 399.6 ± 113.9 microns, respectively. Compared to this pre-endophthalmitis values, there was no statistical difference in the mean CST and MCST at 1, 6, 12 and 24 months, respectively. Figure 2 depicts the CST and MCST values over 2 years.

### Treatment continuation post-endophthalmitis

**Fig. 3:**
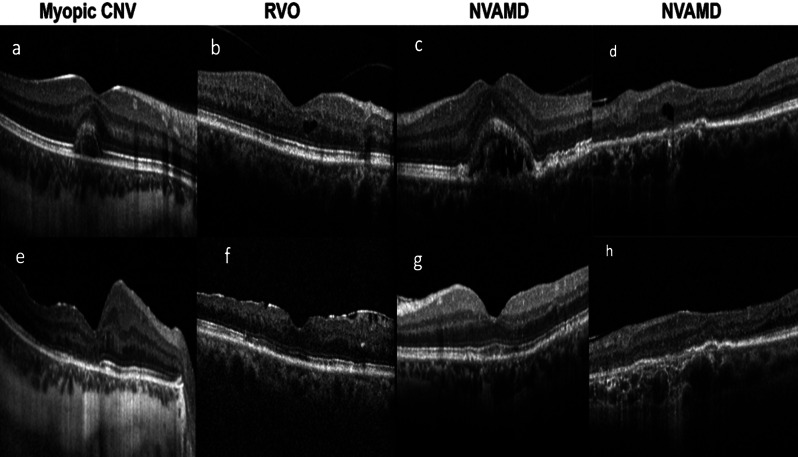
Optical coherence tomography cross sectional scans of the 4 eyes (2 cases of neovascular age-related macular degeneration, 1 case of retina vein occlusion and 1 case of myopic chorioretinopathy) that demonstrated disease quiescence, before (**a**, **b**, **c**, and **d** respectively) and after (**e**, **f**, **g** and **h** respectively) the episode of endophthalmitis

In five out of the eleven patients, treatment with anti-VEGF injections was stopped post-endophthalmitis and this was maintained throughout the 2 years of follow-up. In 4 out of 5, this was due to disease quiescence and in 1 out of 5, it was due to loss of vision. In all 4 cases with disease quiescence, the pathogen was identified in the lab as streptococcus epidermidis, and post endophthalmitis, they demonstrated progressive visual improvement. Figure 3 shows their OCT scans before and after the episode of endophthalmitis. In this singular case of loss of vision, the vitreous sample cultured in the lab did not grow any organism (Table 2).

**Table 2:** Treatment continuation and visual acuity change post-endophthalmitis

No.	diagnosis	Pathogen identified on culture samples	Treatment continuation post endophthalmitis	BCVA ETDRS letters before endophthalmitis	BCVA ETDRS letters at 1 year post endophthalmitis	BCVA ETDRS letters at 2 years post endophthalmitis	Treatment interval before endophthalmitis (weeks)	Treatment interval 1st year after endophthalmitis (weeks)	Treatment interval 2nd year after endophthalmitis (weeks)
1	Myopic CNV	Staphylococcus Epidermitis	Stopped due to disease quiescence	0	65	55	4	NA	NA
2	NVAMD	Staphylococcus Epidermitis	Continued treatment	30	30	0	6	4	3
3	RVO	Staphylococcus Epidermitis	Stopped due to disease quiescence	0	60	60	10	NA	NA
4	NVAMD	No growth	Continued treatment	55	45	35	4	4	5
5	NVAMD	No growth	Continued treatment	0	0	0	4	5	6
6	NVAMD	No growth	Stopped due to loss of vision	0	0	0	8	NA	NA
7	DME	No growth	Continued treatment	70	50	50	4	4	4
8	NVAMD	Staphylococcus Epidermidis	Stopped due to disease quiescence	50	80	85	5	NA	NA
9	NVAMD	Staphylococcus Epidermidis	Stopped due to disease quiescence	55	50	55	4	NA	NA
10	DME	Staphylococcus Epidermidis	Continued treatment	60	60	65	4	4	4
11	DME	Staphylococcus Epidermidis	Continued treatment	65	50	70	4	4	4

BCVA, best-corrected visual acuity; ETDRS, early treatment diabetic retinopathy study; NVAMD, neovascular age related macular degeneration; RVO, retinal vein occlusion; DME, diabetic macular edema; CNV choroidal neovascularization; NA, not applicable

Amongst those 6 patients who continued treatment with anti-VEGF, 2 had extensions in their treatment interval, while 3 stayed on the same treatment interval while it was shortened in the 4th patient.

## Discussion

We observed that only 0.0085% of eyes receiving anti-VEGF injections for retinal pathologies developed endophthalmitis. This incidence rate is notably lower than the 0.056% reported in the meta-analysis conducted by McCannel [16]. Our findings lead us to speculate that the relatively low rates of endophthalmitis in our center may be attributed to two significant factors. Firstly, injections in our center are exclusively administered by ophthalmologists specializing in medical or surgical retina, ensuring a high level of expertise and precision in the procedure. Secondly, we utilize Barraquer speculums with solid blades, which could potentially provide more effective coverage of the lashes and better prevent external contaminants from entering the eye during the injection process.

Overall, the visual and anatomical outcome was worse post-endophthalmitis, then by 2 years, it improved to values similar to the last assessments prior to the infection. However, there were individual variabilities between patients. For example, in 1 eye treated for NVAMD, there was complete loss of vision, and the treatment was stopped post endophthalmitis due to poor prognosis of a significant visual return. However, in this case, the pre-infection visual acuity was very low. On the other hand, there were eyes that showed a significant visual improvement. This kind of variability in treatment outcome, had previously been described amongst NVAMD patients [5]. 

Following the episode of endophthalmitis, 4/11 patients experienced disease quiescence and did not require further treatment with Anti-VEGF agents, up to a period of at least 24 months follow-up. Interestingly in this case, unlike in previous reports that had described this phenomenon in several NVAMD, [5–12] and a case of myopic CNV [11] treated eyes exclusively, we report it in a case of RVO as well. The exact mechanism for this remission remains unclear, and some of the few existing reports have tried to associate the inflammatory process on the retinal landscape secondary to the endophthalmitis, with a plausible alteration in the pathophysiology of NVAMD. For example, in 2019, Kokame et al., [6] suggested that upregulation of guanylate-bonding-protein after endophthalmitis may induce an antiangiogenic state that brings about disease remission in NVAMD. Kally and McCannel, [9] postulated that complement factor H could play a role in NVAMD quiescence. However, given that disease quiescence was observed in non-NVAMD retinal pathologies as well, this indicate that the dysregulation of disease activity may be at the level of VEGF activation, which is the final common pathway for this retinal pathologies-hence their response to anti-VEGF therapy.

A common factor observed amongst all the 4 eyes that experienced disease quiescence is that all vitreous samples were culture positive for staphylococcus epidermidis. These results are similar to that of Kokame et al., [6] who reported disease quiescence after endophthalmitis, in all 7 eyes of 7 patients treated for NVAMD. In Kokame’s series, all the eyes were either infected with staphylococcus epidermidis or culture negative. Staphylococcus and streptococcus infections are the 2 most common causes of endophthalmitis post intravitreal injections, [4] but staphylococcus have been reported to be less virulent than streptococcus [17]. 

Three of the 4 eyes with disease quiescence also underwent vitrectomy. Emoto et al. [8], described 2 cases of post-injection endophthalmitis, treated with vitrectomy and which experienced disease remission with CNV regression. They then postulated that removal of the vitreous gel may have contributed to this disease remission. However, similar findings were reported in non-vitrectomized eyes [6, 9]. 

Limitations to this study, includes the retrospective nature and small sample size. In addition, disease quiescence was determined by fundus exam, OCT imaging and clinical judgement. Fluorescein angiography was not used to assess disease quiescence. However, all 3 cases that demonstrated disease quiescence were followed-up for a period of at least 2 years, and all demonstrated a visual acuity gain, with no worsening of anatomical changes.

In conclusion, we have described the 11 eyes of endophthalmitis recorded in our institution over 16 years of anti-VEGF therapy. Amongst these eyes, 4 out of 11 experienced disease quiescence post endophthalmitis, and this included eyes with NVAMD, RVO and myopic CNV. These results indicate that disease quiescence post endophthalmitis does not occur exclusively in eyes treated for NVAMD and an alteration downstream in the pathophysiology of these retinal pathologies, such as at the level of VEGF activation, seem to be a more plausible explanation. These cases continue to be intriguing and further investigations on the possible mechanism may provide inside into possible future treatment approaches to these diseases.

## Data Availability

No datasets were generated or analysed during the current study.
